# The extrinsic factors important to the homeostasis of allergen-specific memory CD4 T cells

**DOI:** 10.3389/fimmu.2022.1080855

**Published:** 2022-12-15

**Authors:** Aryeong Choi, Yong Woo Jung, Hanbyeul Choi

**Affiliations:** Department of Pharmacy, Korea University, Sejong-si, Republic of Korea

**Keywords:** allergen-specific memory CD4 T cells, homeostasis, chronic inflammatory diseases, microenvironments, extrinsic factors

## Abstract

Memory T cells, which are generated after the primary immune response to cognate antigens, possess unique features compared to naïve or effector T cells. These memory T cells are maintained for a long period of time and robustly reactivate in lymphoid or peripheral tissues where they re-encounter antigens. Environments surrounding memory T cells are importantly involved in the process of the maintenance and reactivation of these T cells. Although memory T cells are generally believed to be formed in response to acute infections, the pathogenesis and persistence of chronic inflammatory diseases, including allergic diseases, are also related to the effector functions of memory CD4 T cells. Thus, the factors involved in the homeostasis of allergen-specific memory CD4 T cells need to be understood to surmount these diseases. Here, we review the characteristics of allergen-specific memory CD4 T cells in allergic diseases and the importance of extrinsic factors for the homeostasis and reactivation of these T cells in the view of mediating persistence, recurrence, and aggravation of allergic diseases. Overall, this review provides a better understanding of memory CD4 T cells to devise effective therapeutic strategies for refractory chronic inflammatory diseases.

## 1 Introduction

Effector CD4 T cells, referred to as helper T (Th) cells, perform protective functions after evasion of distinct types of infectious pathogens, including viruses, bacteria, parasites, and fungi, by enhancing the functions of phagocytes, B cells, or other T cells through the secretion of cytokines (1–3). Th cells, including Th1, Th2, and Th17 cells, differentiate from naïve CD4 T cells after receiving proper cytokines depending on the type of infection or antigen during early immune responses (4, 5). Of particular interest are Th2 cells, because it is known that Th2 cells steer the pathogenesis of diverse allergic disorders by secreting principal type 2 cytokines, including IL-4, IL-5, IL-13, and IL-9 (6–8). These cytokines affect other immune cells, such as B cells, mast cells, eosinophils, as well as non-immune cells, including goblet cells and smooth muscle cells, which drive the pathogenesis of allergic disorders (9, 10).

When antigens are cleared by the immune system, most effector T cells die and only a small portion of these cells survive to serve memory functions. Once these memory T cells re-encounter their antigens, they respond robustly to protect the body by directly killing abnormal cells or further stimulating other immune or non-immune cells. Although these memory functions are generally useful in infectious diseases, the importance of memory CD4 T cells in the pathogenesis of chronic inflammatory diseases, such as allergic or autoimmune diseases, has been documented. For example, the numbers of CD45RO^+^ memory CD4 T cells in the nasal mucosa of patients with seasonal allergic rhinitis increased during continuous exposure to allergens during the pollen season compared to the non-pollen season (11). In addition, memory Th2 cells enhanced the severity of allergic responses, such as eosinophilic lung inflammation and airway hyperresponsiveness, after re-exposure to allergens in experimental allergic asthma models and atopic patients (12, 13).

In addition to their ability to induce robust recall responses, memory T cells retain the capacity to survive antigen-independently and restore their populations using external cytokines from their surrounding microenvironments (14, 15). This characteristic of memory T cells is referred to as homeostasis and is crucial for the maintenance and exacerbation of allergic diseases. Thus, in this review, we summarize the recent findings on allergen-specific memory CD4 T cells and their external homeostatic microenvironments that mediate the pathology of allergic diseases such as allergic asthma, atopic dermatitis (AD), and food allergies (FA).

## 2 The characteristics of allergen-specific memory CD4 T cells in allergic disorders

**Figure 1:**
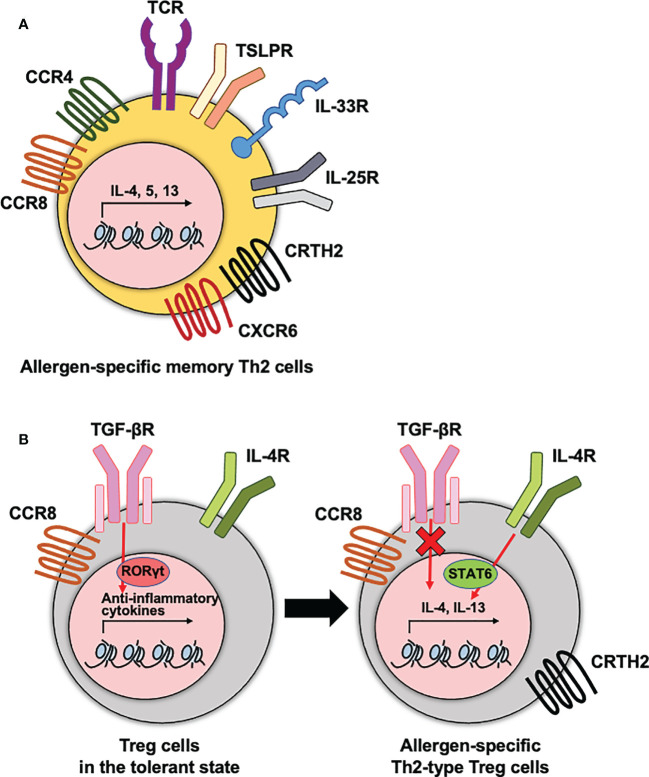
The phenotypes of allergen-specific memory CD4 T cells and Treg cells. **(A)** Allergen-specific memory Th2 cells express diverse cytokine and chemokine receptors that are correlated with the pathogenesis of allergic diseases. **(B)** Treg cells express diverse cytokine and chemokine receptors. To maintain the immune tolerance, TGF-βR needs to be expressed on Treg cells. This receptor signaling mediates RORγt transcription factor for anti-inflammatory functions of Treg cells. In dysfunctional Treg cells, hyperactive IL-4-STAT6 axis and no TGF-β signaling enhances type 2 cytokine production. These receptors contribute to the type 2 cytokine production by Th2 cells and dysfunctional Th2-type Treg cells during the pathogenesis of allergic diseases.

Allergic disorders caused by innocuous environmental antigens manifest aberrant immune reactions in accordance with the exposure of allergens to the epithelium, including the lung, airways, skin, and gastrointestinal tract. Although allergic responses vary depending on the tissue, allergen-specific memory CD4 T cells in barrier tissues orchestrate the pathology of these diseases (**Figure 1A**).

### 2.1 Residency and the functions of allergen-specific memory CD4 T cells in allergic asthma

Allergic asthma is a chronic inflammatory disease of the lung that is continuously exposed to inhaled external materials, including allergens. Th2 cells, major players in the pathology of allergic lung inflammatory disease, accumulated in the bronchoalveolar lavage fluid (BALF) of allergic asthma patients (16). In addition, many studies have highlighted the memory responses of Th2 cells in the pulmonary system in experimental animal models and in asthmatic patients (9, 17–21). These studies provide clues that allergen-specific memory Th2 cells are present in the lungs of asthmatic patients. However, the characteristics of memory Th2 cells have directly not been analyzed for their lifespan and migratory capacity until recently. This was probably due to the proposal that the lung does not provide favorable microenvironments for the survival of memory T cells (22).

Previously, our group employed TCR-transgenic mice and measured the longevity of allergen-specific memory CD4 T cells. We found that these cells survived for over 70 days in the murine lung and airways suffering from allergic inflammation (23). Moreover, it was discovered that allergen-specific memory CD4 T cells showed residency in these tissues and induced rapid and enhanced allergic lung inflammation upon allergen re-exposure. Other groups have suggested that resident memory T (T_RM_) cells in the lung are reactivated by allergens *in situ* and have transcriptional characteristics distinct from those of circulating memory Th2 cells (17, 18, 24). In human asthmatics, allergen-specific CD4 T cells were discovered in BALF after segmental allergen challenge, and these cells mediated allergic lung inflammation by providing type 2 cytokines and eosinophil recruitment (6, 9, 25). Additionally, the microbiota in the upper airways of humans was correlated with the development of adult asthma (26). Contrary to the original proposal, these studies support the notion that allergic lungs provide favorable microenvironments for the homeostasis of allergen-specific memory CD4 T cells.

Recently, it has also been shown that IL-17 producing cells participate in inducing and maintaining allergic asthma. These IL-17-producing memory CD4 T cells were reported to develop from allergen-specific memory Th2 cells after stimulation by proinflammatory cytokines, and to aggravate allergic asthma upon allergen re-exposure in mice and humans (27). Moreover, dual-positive Th2/Th17 cells differentiated from Th2 cells correlated with asthma severity and decreased responsiveness to dexamethasone in the BALF of asthmatic patients (28). These results may indicate that allergen-specific memory CD4 T cells, particularly Th2 cells, mediate disease relapses and aggravation, including steroid unresponsiveness in the lung.

### 2.2 The importance of Th2 and T_RM_ cells in various allergic skin diseases

Allergic skin diseases, such as AD and atopic eczema, induced by protein allergens or chemicals, are multifactorial and complex diseases (29). Similar to asthma, several reports have raised a point that these allergic skin diseases are also considered to be initiated and maintained by CD4 T cells (30–33). Upon allergic stimulations, CD4 T cells were shown to migrate massively into the epidermis of itchy sites and induce type 2 immune responses to mediate allergic skin diseases (34, 35). When allergic eczema patients were rechallenged with allergens, the numbers of allergen-reactive Th2 cells increased, mainly due to the proliferation of these cells in the skin lesions (36). Another study using AD patients presented that allergen-specific Th2 cells persisted in the skin for more than four years (37).

In the case of allergic contact dermatitis and contact hypersensitivity, hapten-specific CD4 T_RM_ cells accumulated even after the skin has healed (38). Further, these cells expanded in the dermis and were sufficient to mediate skin inflammation after re-exposure to the same hapten. Thus, in this type of allergic disease, skin-resident allergen-specific memory CD4 T cells are crucial for mediating local skin inflammation. Altogether, allergen-specific memory CD4 T cells steer the recurrence and exacerbation of allergic inflammation through their persistence and reactivation in the skin.

### 2.3 Th2 responses in FA

FA triggers diverse symptoms by mediating type 2 inflammation in various tissues, including the cutaneous and respiratory systems, as well as the gastrointestinal system. In underlying pathologic mechanisms of FA, allergen-specific IgE and Th2 cells are considered important mediators (39–42). In accordance with the development of research tools, recent studies have indicated that allergen-specific CD4 T cells, in particular, IL-13-producing follicular helper T cells, play a role in the production of high affinity IgE rather than Th2 effector cells (43). These findings highlight that allergen-specific CD4 T cells are also involved in the pathology of FA.

Another important aspect of FA is the breakdown of oral tolerance. Oral tolerance to food antigens is mainly maintained by antigen-specific regulatory T (Treg) cells (39, 44). The activation of STAT6 in these Treg cells stimulated by IL-4 reprogrammed and converted them into a Th2-like phenotype, resulting in an imbalance in oral tolerance (**Figure 1B**) (45). Recently, a greater proportion of activated Tregs and memory-like Treg cells were observed in peripheral blood mononuclear cells (PBMCs) of peanut-allergic patients compared to non-allergic controls (46). Since severe manifestations, such as anaphylaxis, are induced after re-exposure to food antigens, it was speculated that food allergen-specific memory CD4 T cells are present in the gastrointestinal system (47). Although the presence and characteristics of allergen-specific memory CD4 T cells in the gut are yet to be defined, these diseases appear to represent memory responses to food allergens.

### 2.4 The phenotypes of various allergen-specific memory CD4 T cells

There have been many attempts to identify allergen-specific memory CD4 T cells regarding to their functions or disease severities in allergic disorders. Among the markers used to define the populations, cytokine or chemokine receptors expressed on CD4 T cells have well been elucidated as characteristics of allergen-specific memory CD4 T cells and Treg cells that mediate their recruitment and regulate their functions in the tissue (**Figure 1**). These receptors include C-C chemokine receptor 4 (CCR4), a chemoattractant receptor-homologous molecule expressed on Th2 (CRTH2), C-C chemokine receptor type 8 (CCR8), and C-X-C Motif Chemokine Receptor 6 (CXCR6).

#### 2.4.1 CCR4

CCR4 is highly expressed on Th2 cells and contributes to the migration of antigen-specific Th2 cells into inflamed tissue sites (48). In asthmatics, the frequency of CCR4^+^ CD4 T cells was higher in the BALF than in children without any airway diseases (49). Moreover, it has been shown that the frequencies of CCR4^+^ CD45RO^+^ CD4 T cells representing the memory phenotype were higher in allergic patients than in healthy subjects, and that these increased frequencies were positively correlated with AD disease severity (30, 50). In the case of FA, allergen-specific memory CD4 T cells indicated a CCR4^+^ phenotype in the PBMC of shrimp-allergic individuals (51).

#### 2.4.2 CRTH2

The role of CRTH2, which has been used to identify human Th2 cells, includes the production of IL-4, IL-5, and IL-13, chemotaxis of Th2 cells in response to prostaglandin D2, and prevention of apoptosis (52–54). A greater number of CRTH2^+^ CD4 T cells was observed in patients with allergic diseases and was positively correlated with the severity of allergic airway or gastrointestinal inflammation (55–57). CRTH2^+^ allergen-specific memory CD4 T cells are associated with poor compliance to corticosteroids in allergic pulmonary inflammation (58). In addition, a recent study showed that CRTH2 expression on Tregs was also correlated with the malfunction of them as well as enhanced function of Th2 cells in allergic asthmatics (59).

#### 2.4.3 CCR8

The expression of CCR8 has a potential for driving the migration of CD4 T cells including Th2 cells and Foxp3^+^ CD4 T cells into allergic inflammatory sites (60). In atopic asthmatics and dermatitis patients, CCR8-expressing Th2 cells infiltrated in the airways and skin after allergen challenge, respectively (61, 62). Among the compartments of T cells in the skin, CCR8^+^ CD4 T cells are associated with a resident memory phenotype, CD69 and CD103, and greater proliferation capacity in *in vitro* stimulation (63).

#### 2.4.4 CXCR6

CXCR6 is required for the localization of T_RM_ cells in various infection models (64, 65). The level of this gene was highly expressed in the lung CD4 T_RM_ cells than in circulating memory CD4 T cells after allergic lung inflammation (17). Moreover, recent research reported that the CXCR6 expression on Th2 cells correlated with the severity of atopic dermatitis (66).

#### 2.4.5 Others

Rechallenge of allergens increased the expression of diverse cytokine receptors on allergen-specific CD4 T cells. Type 2-associated innate immune receptors, including ST2 (IL-33 receptor) and IL17RB (IL-25 receptor), were highly expressed on allergen-specific CD4 T cells from the BALF of patients with allergic asthma after an allergen challenge (9). In the case of allergic rhinitis, the expression of IL17RB on CD4 T cells also increased in the blood of patients with seasonal allergic rhinitis after exposure to natural allergens (67). Moreover, ST2 expressing lung CD4 T_RM_ cells contribute to the induction of excessive steroid-resistant eosinophilic inflammation in the lung (68).

In conclusion, diverse expressions of chemokine and cytokine receptors on allergen-specific memory CD4 T cells are implicated in the pathogenesis of allergic disorders. Thus, identification of the precise phenotype of allergen-specific memory CD4 T cells will provide a better tool for studying the roles of these cells.

## 3 The extrinsic regulators of allergen-specific memory CD4 T cells in allergic diseases

**Figure 2:**
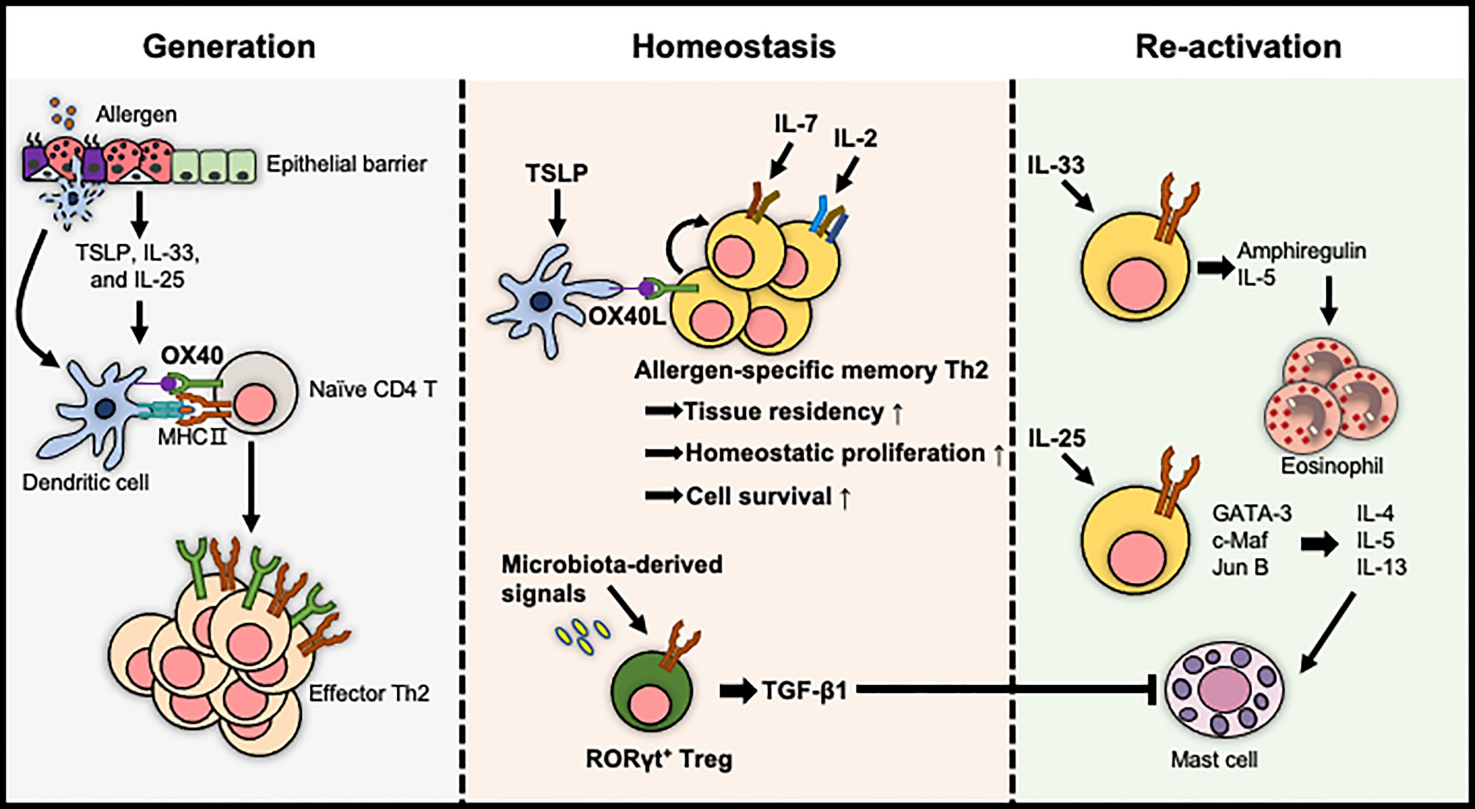
The extrinsic factors for the regulation of allergen-specific memory CD4 T cells. The generation of effector Th2 cells is proposed to be enhanced by the tissue-derived cytokine/DC axis. The pool of allergen-specific memory Th2 cells and their tissue residency are maintained either by cytokines such as IL-2 and IL-7 or by OX40L expressed on TSLP-activated DCs. In contrast, TGF-β1 produced by RORγt^+^ Tregs, which are differentiated by microbiota-derived signals, induces immune tolerance. The reactivation of allergen-specific memory Th2 cells is mediated by IL-33 and IL-25, exacerbating allergic diseases in an allergen-independent manner.

The niches of the immune system regulate the survival, differentiation, and maintenance of immune cells under immune homeostatic or pathological conditions. Memory T cells are antigen-independently maintained, and reactivated by the interactions with neighboring cells, providing exogenous stimuli such as co-stimulatory signals and cytokine signals. In this section, we discuss the importance of exogenous regulatory mechanisms for the reactivation and homeostasis of allergen-specific memory CD4 T cells in allergic disorders (**Figure 2**).

### 3.1 OX40L signalings for allergen-specific memory CD4 T cells

OX40L (CD252), a co-stimulator provided by DCs or IgE-activated mast cells, stimulates allergen-specific CD4 T cells to promote their clonal expansion and survival during the primary response and induces the development of allergen-specific memory CD4 T cells (69–72). Consistent with this, OX40 expressing memory CD4 T cells co-localized with OX40L^+^ DCs or OX40L^+^ mast cells to enhance allergic inflammation in the lung and skin upon re-exposure to allergens or epithelial cell-derived cytokines (73–75). Moreover, a blockade of OX40L induced the tolerogenic state of allergen-specific memory CD4 T cells, resulting in the reduced severity of airway inflammation (75). Altogether, OX40L derived from immune cells, including DCs and mast cells, is crucial for the development, maintenance, and reactivation of allergen-specific memory CD4 T cells to exacerbate allergic diseases.

### 3.2 The tissue-associated signals for allergen-specific memory CD4 T cells

Local tissue-derived cytokines, particularly epithelial cell-derived cytokines called alarmins, such as TSLP, IL-33, and IL-25, are positively correlated with the severity of allergic diseases and are crucial for inducing Th2 polarization (76–79). Furthermore, recent studies have shown that these epithelium-derived signals influence the survival and reactivation of memory CD4 T cells (80–82).

Epigenetic modifications of chromatin structure including DNA methylation and post-translational modifications of histones are well known to determine and maintain the differentiation of cells for regulating the gene expression patterns (83). Moreover, IL-4 in memory Th2 cells is rapidly expressed by this type of epigenetic modification upon antigen re-stimulation (84). Thus, epigenetic changes are crucial strategy for enhancing the function of memory Th2 cells. These alarmins have been proposed to mediate chromatin modification of allergen-specific memory CD4 T cells resulting in the enhancement of type 2 cytokine expressions. For example, TSLP stably programmed the effector state of memory Th2 cells by hyperacetylation within Th2 cytokine locus (81). Additionally, allergen-specific memory T cells expressing ST2 produced high levels of IL-5 by selective remodeling of chromatin structure at the *Il5* locus with a challenge of IL-33 without allergen (85).

Recent research reported another role of IL-33 in allergic response. ST2^hi^ memory CD4 T cells stimulated by IL-33 produced epidermal growth factors, particularly amphiregulin. This growth factor reprogrammed eosinophils toward an inflammatory state and then induced fibrosis of nasal polyps in allergic rhinitis (86).

Although individual alarmins have been intensively studied, these cytokines are also known to be released and to cooperate in the same organ to develop a memory Th2 response. DCs activated by TSLP enhanced the expression of IL-17RB on memory Th2 cells by providing OX40L. Finally, memory Th2 cells, which were stimulated by IL-25, upregulated transcription factors including GATA-3, c-Maf, and Jun B, and type 2 cytokines in the absence of TCR triggering (87). Altogether, these results imply that tissue-derived cytokines have many aspects to reactivate allergen-specific memory CD4 T cells for the pathology of allergic diseases.

### 3.3 The homeostasis of allergen-specific memory CD4 T cells

Increasing efforts to identify homeostatic microenvironments for the formation, survival, and maintenance of allergen-specific memory CD4 T cells have led to a better understanding of the mechanisms underlying allergic disease relapses.

First, it was reported that DCs and IL-2 signalings, which are known to be critical to the activation and proliferation of naïve T cells, contributed to the formation of memory Th2 cells. In a mouse model of allergic asthma, IL-2 signalings provided in the allergic lung microenvironments importantly facilitate the early residency of memory Th2 cells (88). DCs, particularly TSLP-activated DCs, also induced the homeostatic proliferation of allergen-specific central memory Th2 cells to maintain the pool of these cells without antigens (72). Thus, these findings suggest that IL-2- and DC-mediated signalings are crucial for the homeostasis of allergen-specific memory CD4 T cells.

Second, IL-7 and IL-15 are well documented to be important for the survival and homeostatic proliferation of memory CD8 T cells (89). The roles of these cytokines in the maintenance of memory CD4 T cells have also been studied. Between these two cytokines, IL-7 enhanced the survival of memory Th2 cells by increasing the expression of anti-apoptotic protein Bcl-2 (90). This cytokine, produced by Thy1^+^ lymphatic endothelial cells, provided a survival environment for allergen-specific memory Th2 cells in the inducible bronchus-associated lymphoid tissue of the lung, which is formed after allergic lung inflammations in humans and mice (90). IL-7 is also crucial for the development of memory CD4 T cells by mediating the transition of effector T cells into memory T cells (91). In the lung and airways suffered from allergic inflammation, the generation of allergen-specific memory CD4 T cells was affected by IL-7Rα-mediated signals (23). Moreover, in a skin hypersensitivity model, hair follicle-derived IL-7 was required to maintain memory CD4 T_RM_ cells (92). Accordingly, it has been suggested that IL-7 plays an important role in the homeostasis of allergen-specific memory CD4 T cells in barrier tissues.

### 3.4 The control of allergen-specific CD4 T cells

Our bodies have evolved immune homeostasis mechanisms that need to decide between protection against pathogens and tolerance to innocuous substances, such as food, commensal bacteria, and inhaled environmental particles (93, 94). Among these mechanisms, it has extensively examined that Treg cells play a crucial role in preventing the development and aggravation of hypersensitivity against harmless environmental substances (95). The underlying mechanisms include the development of Treg cells in the thymus by TGF-β signaling, which mediates the suppressive functions of these cells in certain peripheral tissues (96, 97). In an experimental model of FA, TGF-β1-deficient allergen-specific Treg cells did not develop into oral tolerance-promoting RORγt^+^ Treg cells, resulting in an increased incidence of FA. Furthermore, the regulation of oral tolerance mediated by RORγt^+^ Tregs was affected by microbiota-derived signals (98). These findings suggest that extrinsic factors contributing to immune tolerance by stimulating allergen-specific CD4 T cells are required to modulate allergic disorders.

## 4 Discussion

Allergic diseases diminish the patient’s quality of life and incur enormous personal and social costs to maintain their lives (99, 100). The roles of CD4 T cells in the steering pathogenesis of allergic disorders have been studied for many decades. In this review, we summarize the characteristics and regulation of extrinsic factors for allergen-specific memory CD4 T cells in hypersensitivity, particularly allergic disorders. Since much lights have been shed to understand the roles of microenvironments in the homeostasis and effector function of allergen-specific memory CD4 T cells, we propose to investigate how these microenvironments can alter the longevity of memory T cells to regulate these diseases. This future direction will guide us to develop a novel strategy for the discovery of therapeutics for allergic diseases.

## Author contributions

Conceptualization: AC, YWJ, HC. Data curation: AC, YWJ, HC. Funding acquisition: YWJ. Supervision: YWJ, HC. Visualization: AC, YWJ, HC. Writing - original draft: AC, YWJ, HC. Writing - review & editing: AC, YWJ, HC. All authors contributed to the article and approved the submitted version.
